# Micro-light-emitting diodes with quantum dots in display technology

**DOI:** 10.1038/s41377-020-0268-1

**Published:** 2020-05-11

**Authors:** Zhaojun Liu, Chun-Ho Lin, Byung-Ryool Hyun, Chin-Wei Sher, Zhijian Lv, Bingqing Luo, Fulong Jiang, Tom Wu, Chih-Hsiang Ho, Hao-Chung Kuo, Jr-Hau He

**Affiliations:** 1 Department of Electrical and Electronic Engineering, Southern University of Science and Technology, Shenzhen, China; 2 School of Materials Science and Engineering, University of New South Wales (UNSW), 2052 Sydney, NSW Australia; 3 Department of Electronic and Computer Engineering, Hong Kong University of Science and Technology, Hong Kong, China; 4 Department of Photonics and Institute of Electro-Optical Engineering, College of Electrical and Computer Engineering, Chiao Tung University, 30010 Hsinchu, Taiwan China; 5 School of Innovation and Entrepreneurship, Southern University of Science and Technology, Shenzhen, China; 6 Raysolution LLC, San Jose, CA 95129 USA; 7 Department of Materials Science and Engineering, City University of Hong Kong, Hong Kong, China

**Keywords:** Inorganic LEDs, Quantum dots

## Abstract

Micro-light-emitting diodes (μ-LEDs) are regarded as the cornerstone of next-generation display technology to meet the personalised demands of advanced applications, such as mobile phones, wearable watches, virtual/augmented reality, micro-projectors and ultrahigh-definition TVs. However, as the LED chip size shrinks to below 20 μm, conventional phosphor colour conversion cannot present sufficient luminance and yield to support high-resolution displays due to the low absorption cross-section. The emergence of quantum dot (QD) materials is expected to fill this gap due to their remarkable photoluminescence, narrow bandwidth emission, colour tuneability, high quantum yield and nanoscale size, providing a powerful full-colour solution for μ-LED displays. Here, we comprehensively review the latest progress concerning the implementation of μ-LEDs and QDs in display technology, including μ-LED design and fabrication, large-scale μ-LED transfer and QD full-colour strategy. Outlooks on QD stability, patterning and deposition and challenges of μ-LED displays are also provided. Finally, we discuss the advanced applications of QD-based μ-LED displays, showing the bright future of this technology.

## Micro-LEDs: A fine display by quantum dots

Micrometre-sized light-emitting diodes (LEDs) based on quantum dots (QDs) will propel the next generation of display technologies, a review by leading researchers shows. Conventional LED designs, with phosphor coatings that convert light to different colours, are difficult to make smaller than 20 micrometres. Jr-Hau He at City University of Hong Kong and co-workers explain how this problem can be tackled using QDs, tiny particles whose optical properties can be tuned by varying their size, providing brighter and more precise colours. Ultra-high-resolution displays based on phospholuminescent QD-LEDs are now being released to the market thanks to finely-controlled methods for synthesising QDs and depositing them onto films. Further research should focus on the best ways to stabilise and protect QD films within LEDs, and to continue developing electroluminescent QD-LEDs, which could potentially outperform their phospholuminescent cousins.

## Introduction

**Fig. 1:**
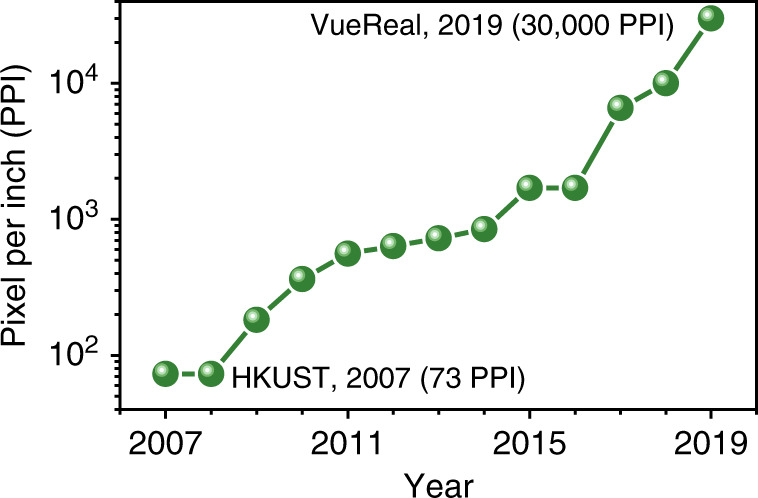
Pixels per inch roadmap of µ-LED displays from 2007 to 2019

Micro-light-emitting diodes (μ-LEDs) have become the focus of display research because of their excellent properties in terms of brightness, lifetime, resolution and efficiency. Over the last decade, the broad prospects for μ-LED applications have attracted a large number of manufacturers. Sony introduced its first 55-inch full high-definition (HD) μ-LED TV panel with 1920 × 1080 resolution in 2012, which consists of over six million individual μ-LEDs. Samsung unveiled the world’s first consumer modular µ-LED 146-inch TV in 2018, which is named “The Wall”. In the academic field, μ-LEDs have been studied for more than 10 years. In 2000, Jiang’s group at Kansas State University started to investigate a microdisk LED with a diameter of ~12 μm^1^. By 2012, the authors had demonstrated prototypes of high-resolution (with 12 μm pixel size and 15 μm pitch size) blue and green microdisplays^2^. Dawson’s group fabricated flexible μ-LED arrays and then performed the first demonstration of visible light communication with a 40-MHz modulation bandwidth and 120-Mbit/s data transmission rate^3^. Lau’s group has been studying microdisplays for more than a decade^4–7^. They reported μ-LED displays with 360 pixels per inch (PPI) in 2012 and 1700 PPI in 2014. Recently, companies further developed ultrahigh-density μ-LED displays to over 10,000 PPI. As the world record, in May 2019, Canadian developer “VueReal” announced μ-LED displays that achieve a density of 30,000 PPI with a high brightness of 100,000 nits. The PPI roadmap of the μ-LED display from 2007 to 2019 is shown in Fig. 1. Despite the rapid development of μ-LEDs, most displays with >1000 PPI are based on a monochrome colour scheme, and the panel diagonals are <2 inches. Currently, it is still challenging to fabricate full-colour and large-scale μ-LED displays with ~2000 PPI, and more effort is necessary to develop mature fabrication for future commercial applications.

Briefly, µ-LEDs are developed based on established solid-state techniques but with more meticulous fabrication processes at a higher resolution than those of regular LEDs. In solid-state LEDs, blue and green LEDs are based on InGaN semiconductors, while AlGaInP semiconductors are used for red LEDs^8,9^. To date, the most efficient violet and blue LEDs based on wide bandgap GaN or InGaN alloys reveal several advantages, such as high efficiency, self-emission (i.e., no extra backlight is required), long lifetime and super-high reliability under harsh environments including high/low temperature, humidity, sunlight and outer space radiation^10^. InGaN-based blue LEDs have reached >80% external quantum efficiency (EQE)^11^, which is the most representative performance indicator of LEDs and is defined as the ratio of extracted photons over injected carriers. Thus, the potential efficiency for a display system that utilises phosphor down-conversion of InGaN LEDs to generate green and red motivates the development of phosphor-converted LEDs (pc-LEDs).

Another commonly used full-colour strategy is employing the combination of red–green–blue (RGB) µ-LED devices in a display. However, compared with the down-conversion technique, this approach has many disadvantages. The first limitation is the low efficiency of green LEDs, which is called the “green gap”. For green LEDs, the active region needs a high fraction of indium, which requires a relatively lower growth temperature (~700 °C), thereby leading to poor crystal quality of the LED epilayer. Furthermore, a high fraction of indium gives rise to a strong polarisation field in the InGaN/GaN multiple quantum wells (MQWs) and results in a strong quantum-confined Stark effect, which decreases the recombination efficiency^12^. Red LEDs are also problematic. The active region of the red LED is composed of AlGaInP material, which possesses a high surface recombination velocity (~10^6^ cm/s)^13^ and, together with a long carrier diffusion length of approximately a few microns, makes the nonradiative surface recombination more significant^14^. Consequently, as the device dimension shrinks to a few microns, the reduced EQE in red µ-LEDs is more serious than that in blue and green µ-LEDs. Another issue in the RGB µ-LED strategy is the drive voltage mismatch between the RGB pixels. The threshold voltage of the blue LED is ~3.3 V, while that of the red and green counterparts is 1.7 V and 2.2 V, which complicates the drive circuit design. In contrast, using blue µ-LEDs with a down-conversion phosphor not only simplifies the driving circuitry but also reduces the number of assembly cycles since the µ-LED arrays can be transferred at the same time. To date, there has been extensive research and development on phosphor materials for pc-LEDs^12^. However, organic or inorganic phosphors are generally not suitable for µ-LED displays due to their spectral broadness and asymmetry, inherent instability, and the fact that the red phosphors suffer from low efficiency, down-conversion energy loss, and low absorption cross-sections in the blue/UV wavelength region^15^. Moreover, the particle size of conventional phosphors may be comparable to or larger than the µ-LED chip, which is problematic.

Recently, colloidal quantum dots (QDs) have appeared as promising emissive materials for replacing organic or molecular phosphors in pc-LEDs^16,17^. QDs are small semiconductor crystals with properties that are vastly different from those of bulk semiconductors as a result of their nanoscale size. The most compelling characteristics of these materials are the tuneability of the semiconductor bandgap by varying their size and discrete energy levels, the so-called quantum confinement effect^18^. The benefits provided by colloidal QDs for LEDs are their narrow emission linewidth (full width at half maximum (FWHM) ~20–30 nm for CdSe- and InP-based QDs, which are the main QD materials in industry and academia), high photoluminescence quantum yield (PLQY > 90%), high photostability, solution processability and low fabrication cost^19^. Intense and saturated colours are obtained at the extrema by narrow spectra, which cover >90% of the strictest Rec. 2020 colour gamut standard^20^. Further, the narrow linewidths of QDs enable them to form viable active elements of LEDs in high-resolution displays. The optical properties of typical phosphors and QDs are summarised in Table 1^21–29^, showing the superiority of QD materials for next-generation display technology.

**Table 1:** Comparison of conventional phosphors and QD materials

Material	Peak emission (nm)	FWHM (nm)	EQE at RT (%)	PL drop at 150 °C relative to RT (%)	References
K_2_SiF_6_:Mn (KSF)	613, 631, 636, 648	4, 4, 3, 3	54.5	≥5	^21,22^
SrLiAl_3_N_4_:Eu	655	52	70	4	^21,23^
Sr_x_Ca_1-x_AlSiN_3_:Eu (SCASN:Eu)	626	86	72	11	^24,25^
CaS:Eu	650	65	53	13	^26,27^
β-SiAlON:Eu	525	45	54	15	^22,28^
SrGa_2_S_4_:Eu	538	46	45	23	^27,29^
Red QD (generation 1)	Tunable	≤35	≥95	30	^21^
Red QD (generation 2)	Tunable	≤35	>95	15	^21^
Red QD (generation 3)	Tunable	<30	≥95	≥1	^21^
Green QD (generation 1)	Tunable	≤35	≥95	30	^21^
Green QD (generation 2)	Tunable	≤35	>95	10	^21^
Green QD (generation 3)	Tunable	<30	>95	4	^21^

FWHM full width at half maximum, EQE external quantum efficiency, RT room temperature, PL photoluminescence

Reproduced from ref. ^21^ with permission from John Wiley and Sons

Currently, liquid crystal displays (LCDs) and organic light-emitting diodes (OLEDs) are the mainstream technologies^30^. LCDs require a backlight source or reflector to generate light propagating through a liquid crystal matrix and colour filters to produce images in colour, but these displays suffer from a low contrast ratio, narrow viewing angle, long response time and high power consumption^31^. In contrast, OLED displays are self-emissive, using a thin film transistor (TFT) backplane to drive each pixel ON and OFF. However, owing to their organic nature, OLED displays have issues in brightness and stability^32^. Additionally, the lower carrier mobilities of organic materials lead to response times in the range of µs^33^. μ-LED displays, as a new promising display technology, offer many potential advantages such as high contrast, wide colour gamut, high speed (~ns) and wide viewing angles^34^. Like OLED, the LED chip in µ-LED displays is no longer just a light source for the backlight module; it plays a role in the direct display of pixel colour and active illumination, achieving the goal of full colour. Incorporation of QDs into µ-LEDs can lead to higher colour rendering and saturation to achieve wide colour gamut requirements and different levels of mixed colour by independently controlling different RGB pixels. In addition, colour compensation can be performed on individual colour pixels (temperature, colour shift, ageing failure, etc.) to achieve a more comprehensive and intelligent high-quality display. Therefore, QD-based µ-LEDs for display applications have recently attracted growing interest owing to their potential advantages over other competitors such as LCDs and OLED displays due to high brightness, low power consumption and favourable cost scaling for large-size displays^35^.

The comparison of different display technologies is summarised in Table 2. In particular, the technological benefits of QD-based µ-LED displays include better colour accuracy, higher colour saturation, higher contrast ratio and higher peak brightness than those of conventional LCDs^36^. Further, compared to OLED displays, QD-based µ-LED technologies have better colour purity (∼100 nm FWHM for OLEDs), higher dynamics range, faster response time, and enhanced lifetime and durability of displays due to higher thermal and air stabilities. Such advantages have made QD-based µ-LED technology ideal for producing displays capable of ultrahigh definition. The superior property of QD materials also makes them attractive to use in other display techniques to improve colour conversion performance. As a result, QD-based displays receive considerable attention in the global market, which was valued at ~1.7 billion USD in 2018 and is projected to increase significantly to 197 billion USD by 2023, with an ~24% compound annual growth rate^37^.

**Table 2:** Comparison of different display technologies

Display Technology	LCD	OLED	µ-LED
Mechanism	Backlight	Self-emissive	Self-emissive
Contrast ratio	5000 : 1	∞	∞
Lifespan	Medium	Medium	Long
Response time	Ms	µs	ns
Operating temperature	−40 to 100 °C	−30 to 85 °C	−100 to 120 °C
Power consumption	High	Medium	Low
View angle	Low	Medium	High
Cost	Low	Medium	High

QD-based display technologies have been mainly developed in two fundamental ways: (i) PL-based LEDs (so-called phosphor-converted LEDs) evolving towards QD-LCD or QD-µ-LED displays; i.e., the QDs are a form of colour-converting film in LCD backlight units, where QDs are physically embedded in polymer matrices sandwiched between two protecting layers or on blue µ-LEDs; and (ii) electroluminescence (EL)-based LEDs leading to QD EL-LED displays. QD EL-(µ-) LEDs have been one of the hot research topics in the QD community and have been extensively explored in recent decades even though their full-scale display commercialisation is not yet in the market. The performance of EL-LEDs is usually benchmarked by EQE. CdSe QD-based EL-LEDs have already exhibited EQE values of 19.8% for blue, 21% for green and 20.5% for red^38–40^, catching up with phosphorescent OLEDs, which exhibit EQE values of ~30%^41,42^. QD EL-LEDs are supposed to outperform all display competitors including OLED displays and even their cousin, QD PL-LED displays. However, QD EL-LEDs are still under development and are not yet in the market. One of the main reasons is the much shorter lifetimes of QD EL-LEDs than of QD PL-based LEDs^43^. With superior performance in terms of extraordinary luminous efficiency, high electro-optical conversion efficiency, and long operation lifetime, QDPL-based displays are the mainstream in the current markets represented by tech giants such as Samsung, Apple, BOE, and TCL. In this review, we focus on the technologies of QD PL-based µ-LEDs and their advanced applications for large-area displays and virtual/augmented reality displays.

## Design and fabrication of µ-LED displays

### LED epitaxy and chip processing

In typical LED fabrication, GaN and InGaN epilayers are usually grown on a c-plane (0001) sapphire substrate or (111) Si substrate by metal-organic chemical vapour deposition (MOCVD)^2,5^. After MOCVD growth, indium tin oxide (ITO) with a thickness of ~100 nm is deposited as a transparent current spreading layer and as an ohmic *p*-contact. Afterwards, the LED mesa is defined through the etching process and is etched down to the n-GaN layer. Then, P and N electrodes are deposited by electron-beam evaporation. In traditional solid-state lighting, the size of LED chips is a few hundred microns, or even millimetres. The manufacturing processes of μ-LEDs are similar to those of traditional LEDs described before, except for the smaller size of the LED mesa. As the size of the LED mesa is reduced to tens of microns, it poses a serious challenge to the LED epitaxy.

**Fig. 2:**
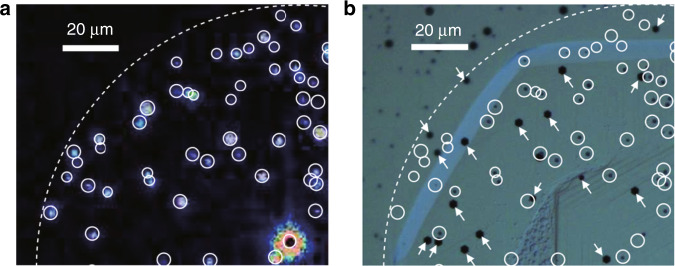
Positional relation between leakage spots and defect states. The emission microscopy measurement shows a strong relation between **a** leakage spots and **b** etch pits (~1 μm size), confirming the importance of defect control during epitaxy. The dashed line and circles indicate the mesa edge and leakage spots, respectively. Reproduced from ref. ^10^ with permission from AIP Publishing

Defectivity is a key parameter for μ-LEDs and significantly affects the display quality and product cost. In the past, the defectivity of the LED mesa was not a concern in the fabrication of conventional LEDs. For example, blue or green LEDs using InGaN/GaN MQWs as the active layers can exhibit high EQE, even with a defect density of ~10^8^ cm^−2^ in the LED epilayer^44^. However, the situation is different for μ-LEDs. Since the defect sizes are usually on the order of μm, when the LED size shrinks, the defects could seriously impact the performance of the device and even dominate the device operation characteristics. It has been confirmed that during epitaxy fabrication, unexpected defects such as dislocations or etch pits cause reverse leakage paths in vertical GaN p-n diodes^10^. As shown in Fig. 2, etch pits (~1 μm size) at the surface of the GaN p-n diode dominantly contribute to the surface leakage current, significantly harming device performance^10^.

Defects with large sizes may even kill the μ-LED devices, resulting in dead or defective pixels, which is not acceptable in μ-LED displays. The control of dead pixels is a major issue for large-scale mass transfer, a typical process in the fabrication of μ-LED displays to assemble a large number of μ-LED units onto a backplane substrate. During the mass transfer process, the transfer block sizes (donor field) for the quad-HD smartphone and 4K TV are 0.92 cm^2^ and 10 cm^2^, respectively, while the killer defect sizes are only ~1 μm and 3 μm, respectively^45^, which means that the defect rate should be <0.1%. Therefore, defect management to control killer numbers during mass transfer is necessary, which is challenging but plays a key role in display fabrication.

**Fig. 3:**
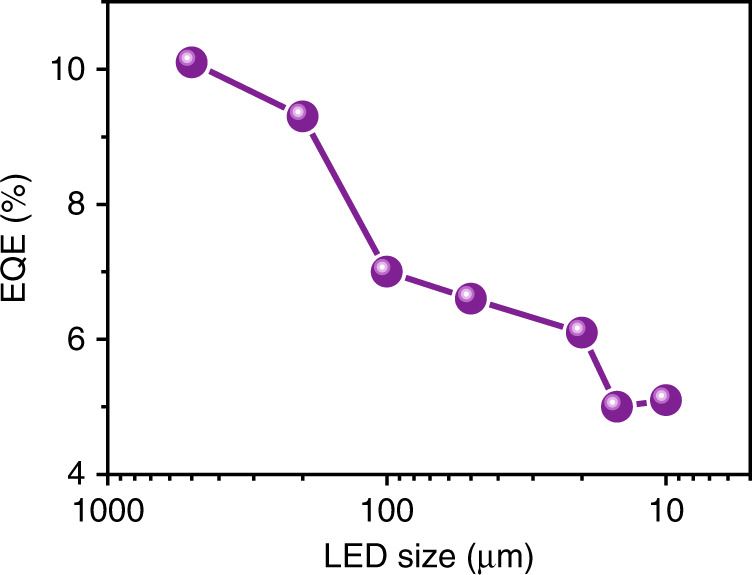
Size reduction effect on μ-LED performance. EQE of GaN μ-LED as a function of LED size. Data compiled from ref. ^46^

Defects can exert far-reaching influences on the performance of μ-LEDs. Generally, the existence of surface defects can generate nonradiative recombination and lead to attenuation of the device EQE. Previous research has found that the performance of LEDs decreases when the chip size shrinks^46^. The EQE versus LED size plot in Fig. 3 shows that the EQE value decreases from ~10% to ~5% as the LED dimension decreases from 500 μm to 10 μm^46^. This phenomenon can be attributed to the larger surface-volume-ratio of the smaller μ-LED, which implies a higher risk of surface defect formation. At the surface, dangling bonds, residual oxides and nitrogen vacancies are possible sources of deep trap states lying in the bandgap region, which act as nonradiative recombination centres, thus decreasing the quantum efficiency. The surface recombination velocity and the carrier diffusion length are two key parameters for surface recombination. The surface recombination velocities of InGaN-based LEDs range from ~3 × 10^2^ to ~10^4^ cm/s, which are far less than that of AlGaInP-based red LEDs (~10^6^ cm/s)^13,47^. Furthermore, the composition fluctuations of InGaN alloys make the ambipolar diffusion coefficients an order of magnitude smaller than that of the phosphide alloy. As a result, the size-dependent efficiency reduction becomes even worse in red μ-LEDs.

**Fig. 4:**
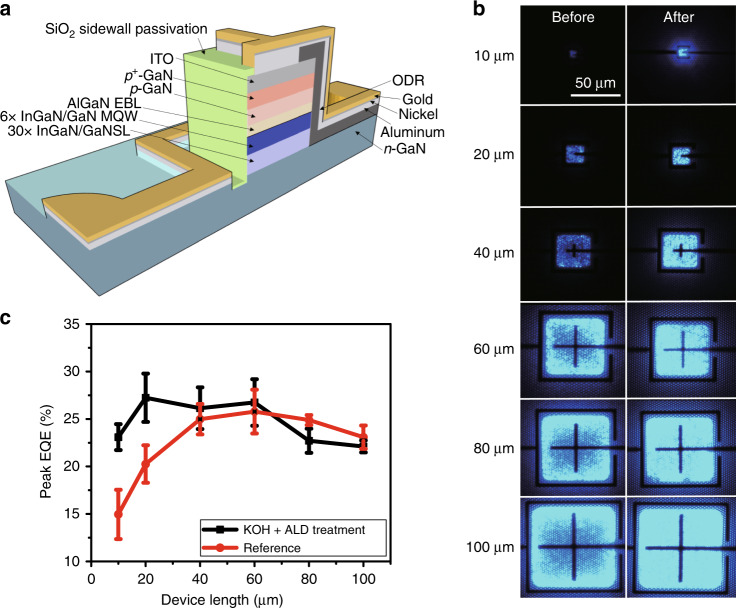
**S**idewall passivation effect in μ-LEDs. **a** Schematic of the μ-LED structure with ALD deposited SiO_2_ for sidewall passivation. **b** Electroluminescence images of the μ-LEDs (sizes from 10 μm to 100 μm) before and after sidewall passivation. **c** The EQE distribution of different device sizes with and without KOH treatment and ALD sidewall passivation. Reproduced from **a**, **b** ref. ^49^ with permission from OSA and **c** ref. ^47^ with permission from Copyright (2019) The Japan Society of Applied Physics

Surface defects not only produce local inferior points but also affect the surrounding area, leading to the performance degradation of entire μ-LEDs, which is called the “sidewall effect”. A previous report has shown that the range affected by surface defects is approximately equal to the carrier diffusion length, typically 1–10 μm^48^. A recent cost-effectiveness analysis showed that to compete with state-of-the-art OLEDs, the size of a μ-LED should be ~3 × 3 μm^2^ for a 5.8-inch 2560 × 1440 resolution quad-HD smartphone and ~9 × 9 μm^2^ for a 55-inch 3840 × 2160 resolution 4K TV^45^. As the range of the sidewall effect is comparable to the size of the μ-LED, surface defects may lead to significant degradation or destroy the whole device. Therefore, surface treatment is necessary to minimise the sidewall effect. It has been confirmed that surface passivation is an effective way to avoid defect states and increase the EQEs of LEDs. Wong et al.^49^ reported that by using the atomic layer deposition (ALD) technique to deposit a SiO_2_ layer for sidewall passivation, the EQE can be effectively improved from 24 to 33% for 20 × 20 μm^2^ μ-LED devices (Fig. 4a, b). Recently, the authors further reported that by using KOH chemical treatment followed by ALD sidewall passivation, the resulting µ-LED devices show size-independent features of the peak efficiency, as shown in Fig. 4c^47^. The EQE of the µ-LED device without surface treatment decreased from 23 to 15% as the device dimensions shrank, whereas the device with sidewall treatment maintained an EQE between 22 and 23%. The results showed that the combination of surface chemical treatment and ALD passivation is an effective way to suppress the sidewall effect.

In addition to surface passivation, there are some general strategies for improving the performance of μ-LEDs. The omnidirectional reflector (ODR) is a frequently used design, as shown in Fig. 4a^49^. By introducing an ODR into the LED structure to reduce the optical loss, the light extraction efficiency can be significantly increased. Another strategy is electrode engineering. Olivier et al.^50^ have shown the EQE improvement of LEDs by replacing the P-electrode with Ag metal, which can lower the contact resistance and result in higher reflectivity.

Spectral uniformity is also an important quality factor for μ-LED displays. To reduce production costs, the use of large wafers is the trend in the LED industry, leading to a significant increase in usable wafer area in each MOCVD process^51^. However, a large wafer may suffer from wafer bowing as a result of a large thermal and lattice mismatch between the LED epilayer and substrate. The major issue is uneven heating on the wafer, which results in nonuniform indium distribution in InGaN/GaN heterostructures during the MOCVD process. A previous study showed that a temperature deviation of 1 °C would induce an emission wavelength variation of 1.8 nm in a blue LED^52^, indicating the importance of temperature control in epitaxial fabrication.

**Fig. 5:**
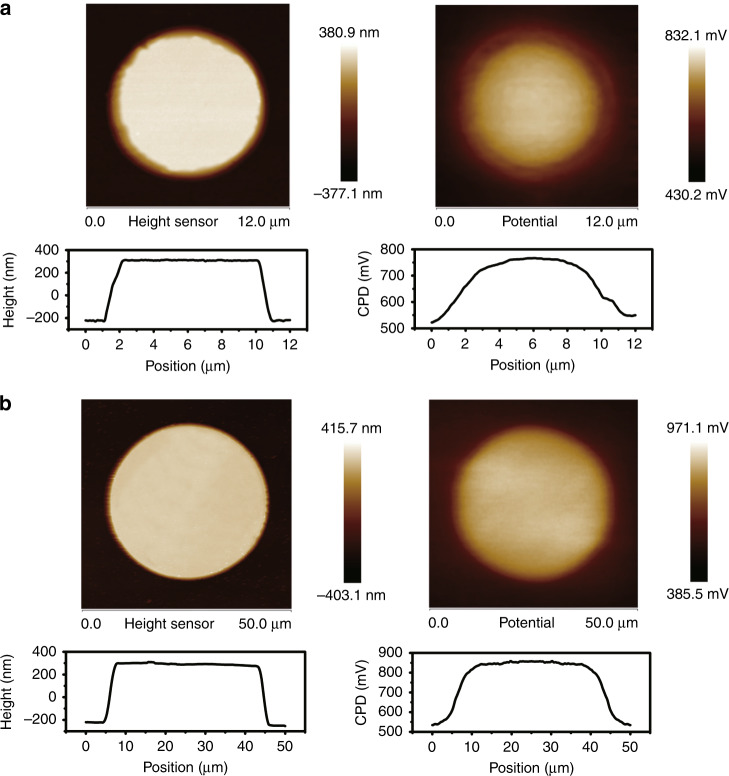
Strain relaxation in small LEDs observed by Kelvin probe force microscopy. Topographic and CPD distribution of μ-LEDs with **a** 10-μm and **b** 40-μm diameters. The central bright region is the LED mesa. Reproduced from ref. ^55^ with permission from OSA

It is worth noting that as the chip size decreases, some challenges for conventional LEDs are overcome, making μ-LEDs beneficial for extensive lighting applications. For example, in the InGaN/GaN MQW structure, the lattice mismatch between InGaN and GaN results in piezoelectric polarisation and produces a strong polarised electric field in MQWs, which is known as the quantum-confined Stark effect^53^. The generated polarised electric field can shift electrons and holes to the opposite sides of a quantum well, thereby reducing the recombination efficiency. In μ-LEDs, size reduction can effectively release the strain and decrease the polarisation field in MQWs. Zhan et al.^55^ have observed strain relaxation in μ-LEDs using Kelvin probe force microscopy (KPFM)^54^ and micro-photoluminescence (PL) techniques. The surface contact potential difference (CPD) of 10 μm and 40 μm LEDs obtained by KPFM is shown in Fig. 5^55^. A much slower CPD slope at the sidewall of 10 μm LEDs shows clear evidence of strain relaxation in the mesa of smaller LEDs.

### µ-LED transfer for large-scale displays

Realisation of µ-LED displays consists of constructing an organised array from a large number of µ-LED units, which are transferred to a receiving backplane substrate for heterogeneous integration into optoelectronic systems. The assembly process for µ-LED displays is based on two technologies. In mass transfer technology, µ-LEDs are separated into individual dice, which are picked up and transferred in groups from a mother wafer to a target substrate. This approach allows spatial distribution of µ-LEDs and is used for fabricating large-size displays ranging from 2 to 70 inches^56^. As another technology, monolithic integration is used to directly hybridise µ-LED chips with a backplate for display assembly. This technology tolerates even smaller pixel pitches of µ-LEDs than the mass transfer approach does. However, as it is critically limited by the wafer size, monolithic integration is mainly applied to build displays with small sizes (<2 inches)^56^.

### Mass transfer

**Fig. 6:**
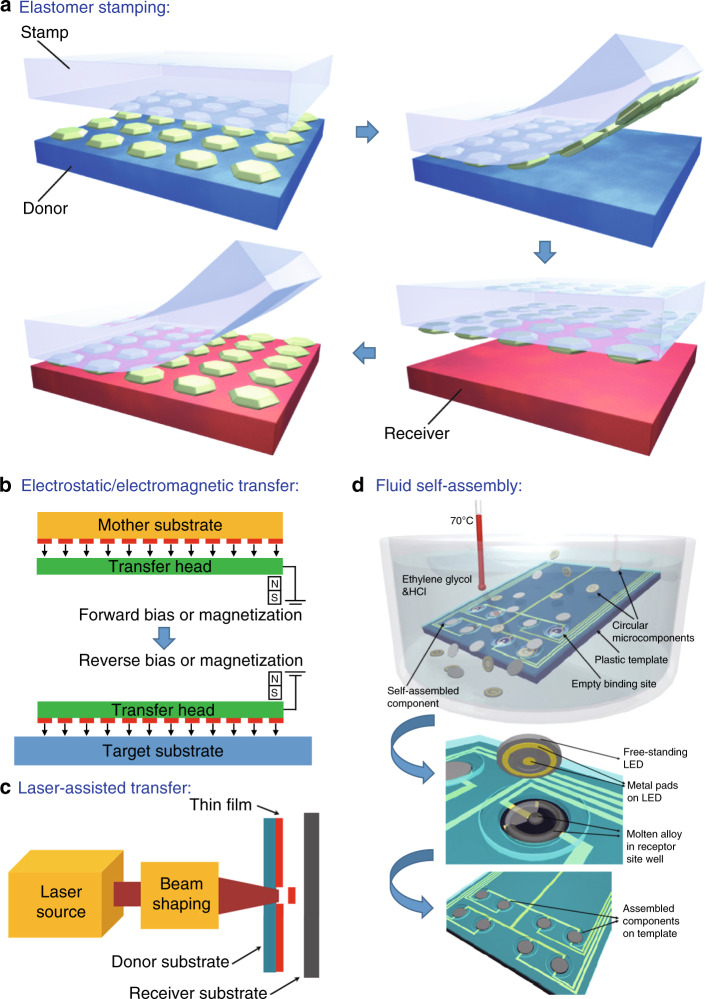
μ-LED mass transfer techniques. Schematics of **a** elastomer stamping, **b** electrostatic/electromagnetic transfer, **c** laser-assisted transfer and **d** fluid self-assembly. Reproduced from **a** ref. ^60^ with permission from Springer Nature, **c** ref. ^70^ with permission from MDPI and **d** ref. ^73^ with permission from IOP Publishing

µ-LED transfer is regarded as one of the most vital fabrication processes for large-scale, high-density and full-colour displays such as TVs, smart phones, wearable displays, virtual reality devices and tablets^57^. Mass transfer is a powerful solution to realise large-scale µ-LED displays because of the ability to transfer over 10,000 µ-LEDs at one time at high speed and low cost. Several approaches have been evaluated to massively assemble µ-LEDs including elastomer stamping^58–62^, electrostatic transfer^63,64^, electromagnetic transfer^65,66^, laser-assisted transfer^67–70^ and fluid self-assembly (Fig. 6)^71–73^.

The elastomer stamping technique has a long history. In 2004, Rogers’s group originally conceived and developed elastomer stamp micro-transfer-printing (μTP) technology, in which devices were transferred by an elastomer stamp utilising the van der Waals force of polydimethylsiloxane (PDMS)^59,60^. The process relies on kinetically controlled switchable adhesion by manipulating the attachment/detachment rate of an elastomer stamp to transfer arrays of micro devices. Based on elastomer stamp μTP, Bower et al.^61^ from X-Celeprint patterned a PDMS stamp with posts for transferring a 20-μm pitch µ-LED array to a 200-μm pitch on a glass substrate. A subset of µ-LEDs is sparsely picked up from dense native arrays by an elastomer stamp with posts. The stamp is used to precisely place the devices onto a target substrate in a dispersed array. The transfer could be repeated several times to populate a full display, successfully demonstrating 10 × 10 mm^2^ colour displays^62^. For continuous stamping, Choi et al.^74^ reported a roll-transfer elastomer printing process for large-scale µ-LED displays. The overlay-aligned transfer of the µ-LED display and single-crystal Si TFT enables the integration of heterogeneous devices on a polymer substrate. The stretchable active matrix (AM) display stably operates under elongation of 40%, indicating a promising route for fabrication of reliable stretchable µ-LED displays.

Bibl et al.^63^ developed an electrostatic transfer method in 2013 by utilising voltage-induced adhesion to transfer µ-LEDs. The transfer head comprises a couple of silicon electrodes over the mesa structure. After contacting the transfer head to the micro devices, a positive voltage was applied to the electrode in the transfer head, creating a grip force on the micro device to lift off the devices from the mother substrate. The exfoliated µ-LEDs were released onto a receiving substrate by applying a negative voltage to the other electrode of the transfer head. Although this technique is very effective in transferring a large number of µ-LEDs at the same time^64^, a high voltage might cause LED breakdown. Therefore, careful control of the voltage is necessary during electrostatic transfer.

In 2016, Wu et al.^65,66^ reported an electromagnetic transfer method that picks up and releases μ-LEDs by varying the magnetic attraction between a transfer head and ferromagnetic layers on μ-LEDs. This process consists of detaching µ-LEDs from the mother wafer with electromagnetic transfer heads, applying an electrical signal to generate a magnetic attraction, and then transferring the µ-LEDs to the receiving substrate. Individual magnetic attraction control of each element enables selective and large-scale µ-LED transfer without any compression process.

Holmes et al.^67^ reported for the first time the use of a laser for the transfer and placement of discrete microparts onto a receiving substrate in 1998. However, laser-assisted transfer did not receive much attention for display fabrication until 2012, when Marinov et al.^68^ developed this technique for selective µ-LED patterning upon laser irradiation on light reactive films. The µ-LED release process is driven by the thermo-mechanical response of the dynamic release layer under laser irradiation. Using a laser, 50 × 50 × 6 μm µ-LEDs were successfully transferred, with an average placement error of 1.8 μm at a high transfer rate exceeding 100M units/h^69^. The high accuracy and fast transfer features have made this method play a key role in µ-LED commercialisation.

Sasaki et al.^72^ demonstrated fluidic self-assembly methods for massive parallel assembly of µ-LEDs in 2017. In the proposed method, µ-LEDs are suspended in a fluid (isopropanol, acetone or distilled water) flowing across an emissive substrate that is designed with wells in the top surface. Due to gravity and capillary forces, the micro devices are driven to move across the surface of the substrate until they are captured in the wells. Precise position and assembly could be achieved during the flowing process, with a transfer rate of over 50 million units per hour^72^.

The aforementioned transfer techniques are summarised in Table 3. The typical elastomer stamping technique could manipulate µ-LEDs at a high transfer yield of 99.99%, while it typically delivers a processing speed of 10,000 to 25,000 devices per hour^61,62^. This means that it would take more than 1 month to assemble a 4K display that is made up of 25 million µ-LEDs. Roll-transfer elastomer printing could be a promising solution to the slow rate^74^. However, the stamp deformation problem compromises the elastomer stamping techniques, leading to poor control and inaccurate registration. Electrostatic and electromagnetic transfer techniques enable large-scale µ-LED transfer, but they may cause LED breakdown due to the application of high voltage and require an additional ferromagnetic layer, respectively. The laser-assisted transfer technique could reach a rate of approximately 100 million per hour with a 1.8-µm placement error yet a low transfer yield of 90%^69^. Fluid self-assembly achieved a high transfer rate of 56 million per hour at low cost^72^, but it still needs further improvement for manufacturing, as modern displays can barely tolerate defective pixels across a screen. To achieve a perfect image of a µ-LED display, some groups have proposed performing individual chip replacements for dead pixels^62^. The other approach is to transfer dual µ-LEDs into each display pixel for illuminance backup^75^. These methods will be either time-consuming or high-cost approaches and thus are insufficient for commercial purposes.

**Table 3:** Summary of µ-LED mass transfer techniques

Elastomer stamping	Electrostatic transfer	Electromagnetic transfer	Laser-assisted transfer	Fluid self-assembly
•Transfer yield 99.99% •10–25K units/h •Small area •Stamp deformation	•Large area •Device break down by high voltage	•Large area •Ferromagnetic layer required	•Transfer yield 90% •100M units/h •1.8 μm placement error	•56M units/h •Low cost

### Monolithic integration technologies

**Fig. 7:**
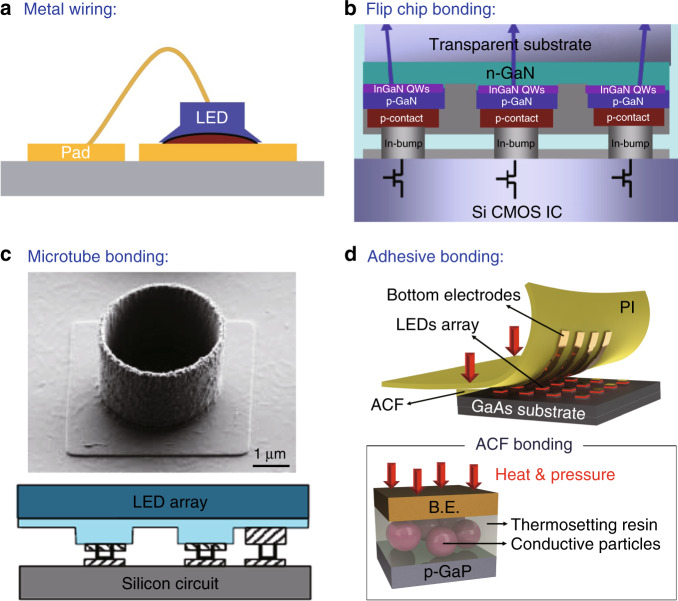
μ-LED monolithic integration techniques. Schematics of **a** metal wiring, **b** flip chip bonding, **c** microtube bonding and **d** adhesive bonding. Reproduced from a ref. ^79^ with permission from Elsevier, **b** ref. ^80^ with permission from AIP Publishing, **c** ref. ^81^ with permission from John Wiley and Sons and **d** ref. ^83^ with permission from RSC Publishing

High-resolution µ-LED displays with a panel diagonal < 2 inches are referred to as “µ-LED microdisplays”^76^. µ-LED microdisplays aim to address applications that primarily require small panels and high luminance, such as see-through glasses, compact hand-held projectors and augmented reality/mix reality devices. For high-brightness microdisplays, the <20-μm small pixel pitch requirement of µ-LEDs cannot be satisfied by mass transfer techniques^77^. Instead, the scaling of µ-LEDs for microdisplays utilises direct integration of µ-LED chips with a silicon backplane at the chip level. To achieve microdisplays with high resolution, µ-LEDs are fabricated on sapphire with the desired small pitch and integrated with the active matrix driving circuit compatible with this pitch. Integration technology relates to the electrical connection of each individual µ-LED pixel to the corresponding pad of the active matrix. Alignment and assembly are important factors for the integration of these two separate parts. Various integration technologies have been investigated, including metal wiring^78,79^, flip chip bonding^80^, microtube bonding^81^ and adhesive bonding^82,83^ (Fig. 7).

Conventional integration techniques such as wiring are simple but limited by low resolution and wire fragility^78^. Jiang and colleagues^80^ used flip chip bonding for display application at a 15-μm pitch. Flip chip bonding is favourable due to reliable integration and high yield but is not compatible with a pixel pitch of 10 μm or less. Both wiring and flip-chip bonding require the assistance of heat, ultrasonic vibration or pressure for the integration, causing device damage and thermal mismatch due to the difference in the thermal expansion coefficient of substrates (sapphire versus silicon). Templier et al.^81^ have already demonstrated the use of microtube technology to integrate heterogeneous devices at a pixel pitch of 10 μm. The microtubes are created on the pads of the circuit and then inserted into the µ-LED pads after alignment. This room-temperature operation overcomes the limitation of the thermal mismatch of the substrate. The resulting displays exhibit brightness up to 1 × 10^7^ cd/m^2^. On the other hand, adhesive bonding typically uses an anisotropic conductive film (ACF) for integration via a thermo-compressive process^84^. The ACF induces strong adhesion and interconnections between µ-LEDs and the bottom electrodes, making it suitable for flexible displays. While significant strides have been made for the integration of µ-LEDs in microdisplays, the scalability of the integration is still a challenge for commercialisation^85^.

### µ-LED plus QD colour conversion for full-colour technology

**Fig. 8:**
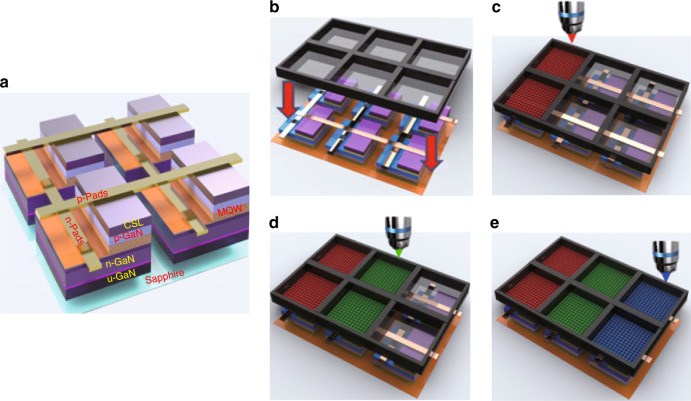
Process flow of the full-colour µ-LED display with photoresist mould design. **a** The structure of the µ-LED arrays. **b** Aligning the mould to the UV µ-LED array. **c**–**e** Consequently jetting the RGB QDs inside the mould window to form the full-colour pixels. Reproduced from ref. ^87^ with permission from Chinese Laser Press

The realisation of QD-based µ-LED full-colour displays mainly relies on RGB chips prepared by QD colour conversion methods^70^. In 2015, for the first time, Han et al.^86^ developed aerosol jet printing to spray RGB QDs on the surface of a UV µ-LED array to achieve a full-colour µ-LED display. The wavelength of the excitation source was 395 nm, and the size of the UV µ-LED chip was 35 µm × 35 µm. This study demonstrated a full-colour QD-converting display and laid an excellent foundation for subsequent research. One of the unresolved issues in Han’s study was the optical crosstalk (32.8%) between the RGB QDs, which resulted in a fuzzy boundary between adjacent pixels. Later, in 2017, Lin et al.^87^ successfully reduced the optical crosstalk by inserting a black photoresist mould between pixels, which consists of a window for QD printing and a blocking wall, as shown in Fig. 8. The use of a photoresist mould structure dramatically reduced the crosstalk value to nearly zero, thus showing great potential for the fabrication of high-quality full-colour µ-LED displays.

One issue in QD colour conversions is how to completely remove the blue excitation light from GaN-based LEDs. One approach is employing a distributed Bragg reflector (DBR) structure on top of QD layers to avoid leakage of blue light^86–88^. Another way is to deposit/print colour filters, which are also known as the fundamental structure of TFT-LCD modules, consisting of three main colours: red, green and blue^89^. However, both methods require more fabrication processes and simultaneously increase the production cost. As the simplest way, QDs themselves can be used to absorb blue light due to their high absorption in the wavelength region below 450 nm. Recently, a simulation using QD extinction coefficients and the QD volume fraction in a QD-polymer solution demonstrated that green and red QDs can achieve 99% absorption of blue light with a QD film thickness of 5 µm and a certain volume fraction^90^. Additionally, it was reported that an inkjet-printed InP/ZnS QD film absorbs more than 95% of blue light when the film thickness is ~10 µm^91^. Further, the reliability test showed only minimal degradation under high-temperature and high-humidity conditions (65 °C and 95% relative humidity). These studies reveal the potential of QD films for high absorption applications. However, to achieve the goal of full extinction of blue light and reliable QD films, further optimisation of the QD concentration and film thickness should be conducted in the future.

Researchers have also developed several strategies to improve the efficiency of QD-based µ-LED displays. Chen et al.^88^ fabricated an RGB µ-LED display with red and green QDs excited by a 451-nm blue µ-LED array. In this display, a hybrid Bragg reflector (HBR) was deposited on the bottom of the substrate to reflect the RGB light, and the resulting output intensity was enhanced by 27%. The nonradiative Förster resonant energy transfer (FRET) mechanism^92^ has also been implemented in µ-LED displays to improve the colour conversion efficiency^93,94^. The basic idea is to transfer excess excitons generated in InGaN/GaN MQWs to QD acceptors, thus producing more light from QDs. Three conditions must be met for FRET: (i) the absorption spectrum of the acceptor must overlap with the emission spectrum of donors, (ii) the transition of the dipole orientation should be parallel, and (iii) the transfer path between donor and acceptor excitons should be sufficiently small (typically <10 nm)^95^. Therefore, it is necessary to make the gap between QDs (acceptor) and MQWs (donor) as short as possible in the µ-LED.

**Fig. 9:**
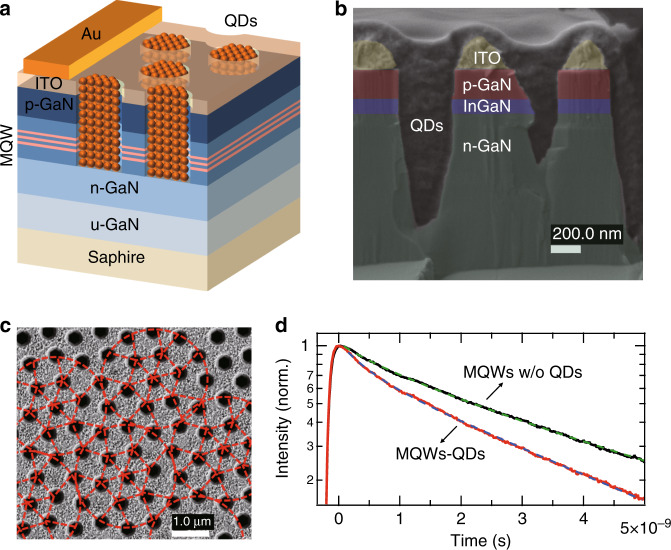
Förster resonant energy transfer nanohole design in LEDs. **a** Schematic representation, **b** cross-sectional, and **c** top scanning electron microscope images of a photonic nanohole LED hybridised with QD colour converters. **d** Time-resolved photoluminescence decays of LED with pure MQW (black line) and MQW-QD contact (red line) structures. Reproduced with minor editing from ref. ^96^ the Optical Society under the terms of the Creative Commons Attribution 4.0 License

To reduce the gap between QDs and MQWs, Krishnan et al.^96^ fabricated periodic nanoholes on the surface of an LED chip and deposited QDs into the nanoholes, making the QDs directly contact the MQWs, as shown in Fig. 9. Based on the FRET strategy, the world-record effective quantum yield (QY) of 123% was achieved^96^. The transient PL traces reflect the lifetime of excitons in a quantum well, and the fast PL decays corresponding to PL quenching in the static emission spectrum of MQWs can be considered clear evidence of exciton transfer from MQWs to QD acceptors^92^. Compared with the PL decay in the pure MQW structure, the measured PL traces in the MQW-QD contact structure showed faster PL decays, as expected by FRET (Fig. 9d)^96^. Hybrid III-nitride/QD-nanohole white LEDs fabricated by Zhuang et al.^97^ also demonstrated a high colour conversion efficiency of 69% and a high effective QY of 93%, which were attributed to efficient FRET between MQWs and QDs, with the FRET efficiency reaching 80%. Additionally, the optimisation of white emission indexes by tuning ternary complementary colours led to hybrid LEDs with a high colour rendering index of 82, which covers the white light emission at correlated colour temperature (CCT) ranging from 2629 K to 6636 K. Instead of the nanohole structure, Liu et al.^98^ demonstrated a colour conversion efficiency enhancement by depositing QDs on nanorod-shaped MQWs. Compared with its unstructured counterpart, the resulting LED exhibited a 32.4% enhancement of colour conversion efficiency, demonstrating the superiority of FRET design.

**Fig. 10:**
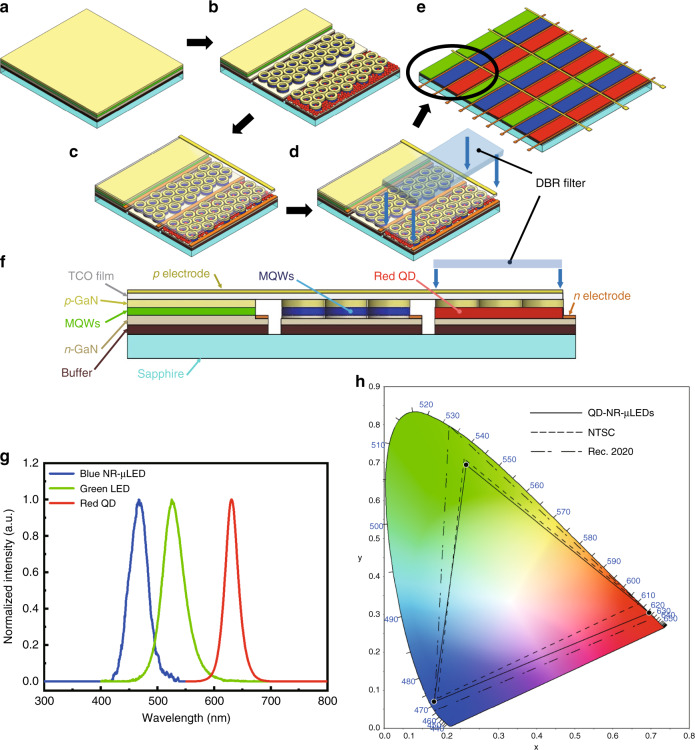
Full-colour QD-NR μ-LED display design and performance. **a** Epitaxial wafer. **b** Three subpixels of a green μ-LED, a blue NR μ-LED, and a red QD-NR μ-LED. **c** Deposition of transparent conducting oxide film and p-n electrodes. **d** Covering DBR filter. **e** Full-colour QD-NR μ-LED display. **f** Cross-sectional view of a single RGB pixel. **g** EL spectra of a QD-NR μ-LED display. **h** Colour gamut of a QD-NR μ-LED display, NTSC, and Rec. 2020. Reproduced from ref. ^101^ with permission from Chinese Laser Press

A novel nanoring (NR) structure was also proposed in 2017 to improve the FRET effect^99^. For nanohole and nanorod structures, either the inner or outer sidewall is exposed to contact with QDs. However, for the NR structure, both sidewalls contact QDs, which can significantly improve the colour conversion efficiency. Wang et al.^99^ have shown NR-LED arrays with a diameter of <1 μm on a green epitaxial wafer, and the emission wavelength of the NRs can be tuned from green (535 nm) to blue (480 nm) by changing the NR wall width. In addition, due to nanoscale strain relaxation^100^, the quantum-confined Stark effect is reduced in GaN-based LEDs with the NR structure. Figure 10a–f shows the fabrication of a full-colour hybrid QD-NR μ-LED display by Chen et al.^101^. Intriguingly, ternary complementary colours were achieved based on different mechanisms. The display used a green LED epitaxial wafer as the base to emit bright green light. Then, the NR structures were constructed on the green LED, and the colour of NR μ-LEDs was adjusted from green to blue by controlling the wall width of the NRs. Using the ALD technique, a 1-nm Al_2_O_3_ layer was deposited on the sidewall of blue NR μ-LEDs for total internal reflection, improving the emission intensity of NR μ-LEDs by 143.1%. Finally, the red QDs coupled with MQWs were sprayed onto blue NR μ-LEDs for the full-colour display. To further enhance the brightness of the red emission, a DBR in blue wavelength was employed to reuse the excitation blue light. The LED chip size was 10 μm × 10 μm, while the RGB subpixels were 3 μm × 10 μm. For such small subpixels, the aforementioned aerosol jet printing would no longer be applicable. Thus, red QD spraying on NR μ-LEDs was implemented using a super-inkjet printing system to accurately spray the QDs on the subpixel area, giving a linewidth of 1.65 μm and a thickness of 56.3 nm for the QD patterns. The emission spectrum and CIE gamut of the QD-NR μ-LED display are shown in Fig. 10g, h, respectively^101^. The colour gamut of the display can achieve 104.8% NTSC and 78.2% Rec. 2020, which is sufficient to support full-colour performance for practical applications.

## Outlook of µ-LEDs and QDs

### QDs for µ-LED and their stability

For full-colour display solutions, conventional RGB colour conversion chips have inadequate luminance and low yield issues when the LED chip size is <20 μm^15^. The emergence of QD materials is expected to fill this gap. Many colloidal QD materials have been studied over the past few decades for their potential use in display applications. However, only CdSe- and InP-based QDs have achieved sufficient display performance for translation to industrial manufacturing^102^. InP QDs are considered the most promising candidates for heavy metal-free QDs, with emission colours covering most of the visible and near-infra-red window^103^. Their weaknesses such as lower QY and broader FWHM are typically attributed to the surface defects caused by incomplete shell coverage or interfacial strain because of lattice mismatch between the InP core and the conventional shell material^102,104^. Nevertheless, the qualities of InP QDs have been steadily improved by growing thicker shells, reducing lattice strain^102^. In contrast, recent progress in QD synthesis has led to CdSe-based QDs (core/shell or alloyed QDs) with favourable properties, including a high QY of >90%, narrow FWHM of ~20 nm, and thick shell with a thickness of >10 nm, yielding dramatically improved stability, while the state-of-the-art InP-based QDs have not yet reached such high quality^19,36,102^.

**Fig. 11:**
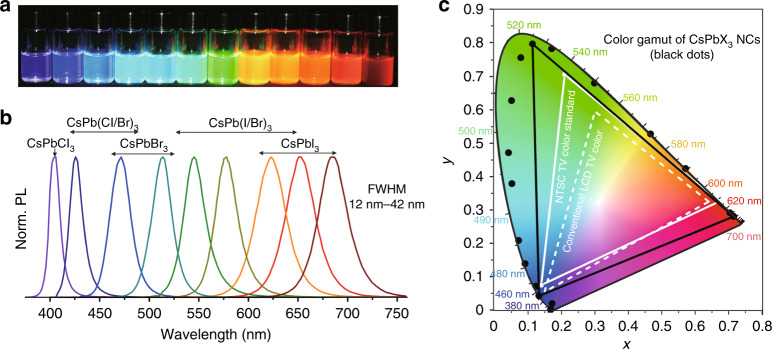
Perovskite QDs for wide colour gamut conversion. **a** Colloidal CsPbX_3_ (X = Cl, Br, I) QD solutions in toluene under a UV lamp and **b** their representative PL spectra. **c** CIE chromaticity coordinates (dark points) from the emissions of CsPbX_3_ QDs compared with those of commercialised LCD TVs (dashed white line), reaching 140% of the NTSC colour standard (solid white line). Reproduced from ref. ^112^ with permission from ACS Publications

Recently, halide perovskites with the general formula of APbX_3_ (A = CH_3_NH_3_^+^, Cs^+^, FA^+^, or mixture; X = I^−^, Br^−^, Cl^−^) have become an emerging class of colloidal QDs for next-generation displays^105^. The popularity of perovskites originates from their ultrahigh photovoltaic efficiencies of up to 25.2%^106^, which is attributed to their exceptional optical and transport properties, such as high absorption over the visible spectrum^107^, low exciton binding energy^108^, charge carrier diffusion lengths in the µm range^109^, and high defect tolerance^110^. The rapid advances in bulk halide perovskites have in turn brought significant attention to perovskite quantum dots (PQDs)^111–113^. PQDs possess many favourable features for LED applications, including near-unity PLQYs (>90%) without a shell layer, narrow-band PL (FWHM ~12–40 nm), and easily tuneable emission wavelengths from ultraviolet to near-infra-red by either controlling the halide composition or QD size^114–116^. Additionally, their narrow spectral bandwidths lead to a wider colour gamut encompassing 140% of the NTSC standard (corresponding to 93% coverage of the Rec. 2020 colour gamut standard) and covering the CIE 1931 colour spaces (Fig. 11)^112^. Inspired by this result, researchers incorporated RGB PQD films into an LCD backlight, and an ultrahigh colour gamut of over 100% of Rec. 2020 standard was achieved^117,118^. The excellent luminescence and charge transport properties have invigorated enormous research efforts on PQD-based light-emitting devices in display and solid-state lighting applications. In the academic field, PQDs have been mostly applied as active layer components in EL-based light-emitting devices. The initial low efficiency of EL-based PQD-LEDs arises from multiple physical mechanisms, such as poor optimisations of device structure, high leakage current due to poor morphology, significant nonradiative recombination at the interface, and charge injection imbalance. These problems have been quickly solved. In 2018 and 2019, tremendous progress in the efficiency of EL-based PQD-LEDs has been reported, with EQE values of over 20% for green and red colours^119–121^.

Now, we divert our attention to PL-based PQD-LEDs, which are closer to commercial applications than are EL-based devices. PQDs are very attractive materials for QDPL-based µ-LEDs because of their excellent optical and physical properties as mentioned above. With superior colour conversion capability to generate RGB lights, PQDs have been utilised in PL-based white LEDs, and ultrahigh luminous efficiency >100 lm/W (the standard for practical use in room lighting)^122^ has been achieved. For example, Zhou et al.^123^ developed PQD-embedded polymer composite films for colour conversion, and the resulting white LEDs revealed a luminous efficiency of 109 lm/W and a wide gamut of 121% NTSC. Yoon et al.^124^ also demonstrated the encapsulation of PQD powder with a mixed silicon nitride for white LEDs, which showed a luminous efficiency of 138 lm/W (EQE = 51.4%), colour gamut of 128% NTSC and CCT of 6762 K. Due to their impressive performance, PQD-based displays were further developed by manufacturers. At the exhibition of SID Display Week 2019, a display manufacturer CSOT demonstrated a 6.6-inch 384 × 300 LED display that uses PQDs for colour conversion, confirming the potential of PQDs for next-generation lighting applications.

However, the most serious concern for any QD colour conversion films, including CdSe, InP, and perovskite QDs, in commercialisation is their instability, which leads to PL quenching arising from the degraded QD materials (in colour conversion film). Therefore, understanding the degradation mechanism and improving the stability of QDs have become hot topics in the QD materials community. Many degradation mechanisms in PQDs and their LED devices are similar to those of CdSe-/InP-based QDs^43^. The QD degradation mechanisms have been categorised into structural stability, interfacial stability, atmospheric stability (by light, oxygen and moisture) and thermal stability^125^. Enormous efforts have recently been made to overcome the instability of perovskite materials including PQDs in solar cells and LEDs^126–129^. In particular, there are four general strategies that can be applied to all QD materials to improve their stability: (i) increasing the mechanical stability of the crystal structure by ion doping, (ii) performing surface engineering by controlling ligands, which passivate defect states and produce strong binding motifs on the QD surfaces, (iii) encapsulating QD layers with polymers and oxide materials, and (iv) performing more efficient packaging of devices^125^.

**Fig. 12:**
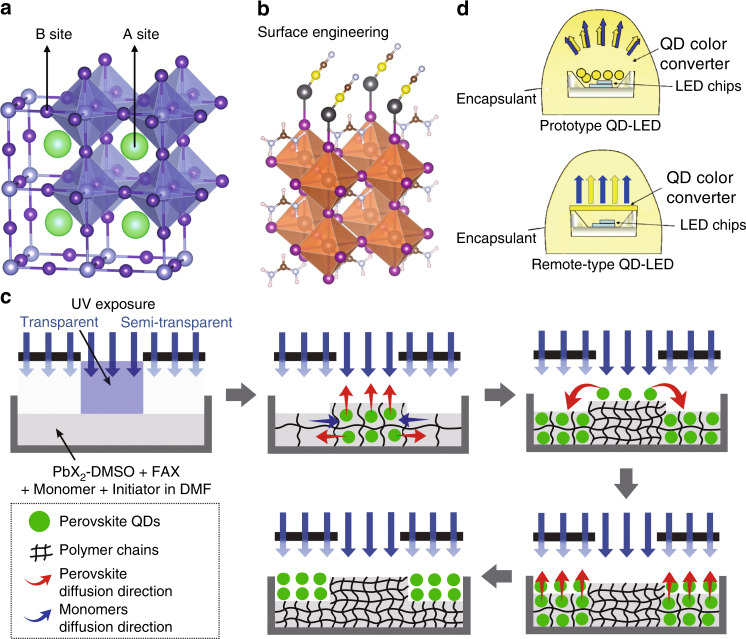
Strategies for improving the stability of perovskite QDs. **a** A-site or B-site doping. **b** Surface engineering. **c** Encapsulation with a polymer or oxide matrix. **d** More efficient device packaging (i.e., remote-type design can enhance the stability of QDs compared with the prototype structure). Reproduced from **a** ref. ^132^ with permission from RSC Publishing, **b** ref. ^134^ with permission from AAAS, **c** ref. ^137^ with permission from John Wiley and Sons, and **d** ref. ^125^ with permission from RSC Publishing

Taking PQD as an example, A-site (Cs^+^, FA^+^, PEA^+^, etc.) and B-site (Mn^2+^, Ni^2+^, Ln^3+^, Sn^4+^, etc.) doping on perovskites has been carried out to improve the material stability (Fig. 12a)^130–132^. The appropriate doping can generate suitable host–guest interfaces to passivate unwanted trap states and inhibit nonradiative recombination, leading to enhanced stability under ambient conditions^133^. Surface engineering can significantly improve the stability of PQDs (Fig. 12b)^134^. Generally, during PQD synthesis, the PQD is capped by long alkyl oleic acid (OA) and oleylamine (OLA), which are not tightly bound to the PQD surface and make PQD unstable^135^. Using controlling ligands to construct strong binding motifs on the PQD surface can be a good solution. For example, Li et al.^136^ utilised stearic acid and octadecylamine to replace OA and OLA, and the degradation of PQDs was suppressed from 60 to 20% after 30 days. Polymer or oxide encapsulation is a very straightforward method that directly protects PQDs from oxygen and moisture in the air (Fig. 12c)^123,124,137^. However, due to the highly ionic properties of PQDs, the use of nonpolar encapsulation materials is important to avoid undesired interactions between PQDs and the encapsulation matrix^138^. Efficient packaging of LEDs also plays a key role (Fig. 12d)^125^. For example, it was reported that by using remote-type design (i.e., QDs are dispensed on top of the LED package)^139^, the stability of PQDs can be notably improved compared with that using the conventional prototype structure (i.e., QDs are dispensed inside the LED package)^140^. Although many strategies have been utilised, PQDs have not met the stability requirement for practical use, and more research efforts are still necessary.

Currently, the combination of ligand cross-linking and polymeric encapsulation has been widely used to protect CdSe-/InP-based QDs in commercial QD displays. Moreover, it is well known that QD shell layers are very critical to enhance the QD stability as well as the device performance, such as colour purity, quantum yield, and efficiency roll-off. Type I core/shell QDs are commonly used in QD-based LEDs, in which the core QDs are overcoated with wide bandgap shell materials that passivate surface defects and confine excitons to the core, thus increasing the PLQY of the QDs^39,141^. However, the increased PLQY does not guarantee improved LED performance. In particular, the efficiency drop by the roll-off effect is the main bottleneck to most types of QD-based LEDs. The physical origin of the roll-off effect in QD-based devices is still controversial. Generally, it is believed that this effect comes from nonradiative Auger recombination by charged and neutral multicarrier states in QDs^142,143^. In particular, when the pixel size of PL-based QD µ-LEDs decreases to a few tens of microns, the illumination from µ-LEDs should increase accordingly to obtain enough brightness, which makes the PL quenching from Auger recombination of multicarrier states more serious. To suppress Auger recombination, core/shell QDs with thick shells or graded alloyed shells have been successfully used by Klimov’s group to demonstrate the high thermal stability, droop-free efficiency, and colour purity of QD-based LEDs^144,145^. Further, for µ-LEDs using on-chip design (i.e., QDs are directly dispensed on top of µ-LEDs), the thermal stability and lifetime of QDs could be a major concern due to the high flux and high LED contact temperature (∼150 °C)^146^. Currently, in state-of-the-art CdSe-based QDs, shell layers can protect the core QDs and maintain their high PLQYs (>95%), even at temperatures > 150 °C^147^. In a recent work, Shimizu et al. demonstrated a performance improvement (~10%) of white LEDs by combining Cd-based QDs with a conventional phosphor material^148^. The reliability test under high temperature (95 °C for 3000 h) and high humidity (90% relative humidity for 1000 h) showed that the white LEDs meet the commercial market requirements. Compared with that of CdSe-based QDs, the development of PQDs is still in the infancy stage, and their stability is not sufficient. Thus, more research on core/shell PQDs with a thick shell or a graded alloyed shell is urgent for PQD-based µ-LEDs. To date, the stability of QDs engineered by various approaches ranges from a few hours to several months under ambient conditions or UV exposure^43,125^. However, the general requirement for commercialised displays is >10,000 h, which means that there is still a long way to go to incorporate QDs in extensive commercial applications.

Finally, we briefly discuss the toxicity of QDs. It has been reported that heavy metal-containing QDs have been shown to be toxic in human and environmental models^149^. One of the major obstacles to the commercialisation of QD-based optoelectronic devices arises from product legislation such as the Restriction of Hazardous Substances (RoHS), which is a directive regulating the manufacture, import and distribution of electronics and electrical equipment within a country. After the first RoHS (Directive 2002/95/EC) was published by the European Union (EU) in 2002, many countries started to enact their own versions of legislation RoHS. For example, EU RoHS specifies maximum levels of substances such as <100 ppm Cd and <1000 ppm Pb (by weight). The higher limit of Pb makes PQDs appealing candidates for replacing Cd-based QDs in display applications. Ultimately, the use of heavy metal-free QDs could be the best option in any QD application. However, the performance of heavy metal-free QDs does not reach the levels of Cd-containing QDs or PQDs for display applications, and further study is needed to improve the QD quality.

### QD patterning and deposition

Typical QD-converted µ-LED displays require the deposition of red and green QDs on blue µ-LED chips. Conventional QD deposition methods, including spin-coating, phase-separation, self-assembly, dip-coating, and layer-by-layer deposition, have many limitations for fabrication, such as complicated and multiple steps, low resolution, and difficult patterning control. Additionally, these techniques usually need a large amount of QDs in solution form but actually transfer a very limited amount, thereby considerably increasing the production cost. Therefore, advanced techniques for patterning QDs on µ-LEDs have been proposed, such as photolithography^150^, electron-beam lithography^151^, jet printing^86^, 3D printing^152^, microcontact printing^153^, and dip-pen nanolithography^154^ methods. To select a suitable QD patterning technique, the resolution, throughput, and defect tolerance of the desired display should be considered. For example, in 55-inch 4K (3840 × 2160 resolution) TV, the size of the µ-LED chip is ~10 µm × 10 µm, which consists of small-size RGB subpixels ~3 µm, and a total subpixel number of 24,883,200 is required to construct the entire TV display. Further, high-resolution displays such as 8K TV or virtual reality (VR)/augmented reality (AR) displays demand even smaller subpixel sizes (<3 µm) and much higher subpixel numbers.

Generally, using electron-beam lithography, QD films with patterns scaled down to sub-µm can be achieved^151^, which is good enough for normal µ-LEDs. In addition, using hydrogen silsesquioxane resist with precise parameter control, Manfrinato et al.^155^ reported nanometre-resolution patterning based on electron-beam lithography. The reported sizes of QD patterns by photolithography are 2–5 µm in width and ~50 µm in thickness^150^. With printing techniques, 250 nm QD pixel resolution was demonstrated by Richner et al.^156^. The dimension characteristics of various deposition methods are summarised in Table 4. Thus, the pixel and pitch resolution is not an issue for QD film deposition on µ-LEDs by using any techniques mentioned as long as the LED size is in the range of µm. However, each technique has its own limitations. The microcontact and transfer printing approaches need a template, making the procedure more complicated. Inkjet printing has problems such as easy clog of the jagged nozzle, the coffee ring effect, and nonuniform film thickness and rough surface of the printed film, even though it is the most desirable manufacturing process in the display industry. In the QD deposition process with photolithography and electron-beam lithography techniques, QD films could be easily damaged, and the production costs are high.

**Table 4:** Comparison of different patterning techniques for QD deposition

Patterning technique	Pattern linewidth	QD film thickness
Photolithography	Several µm	Several µm
Electron-beam lithography	Several nm	~10 nm
Inkjet printing	250 nm	Several µm
Microcontact printing	Several dozens nm	Monolayer
3D printing	A few µm	N/A

So far, printing technology can satisfy most of the demands because the pixel sizes of displays are still above microns in the current market. From the viewpoint of simple and rapid fabrication, printing techniques should be mainstream in the future display industry. However, the problem of nonuniform printing should be further addressed. Developing a stable, high-resolution, and cost-effective process that can directly print uniform QD layers with diverse sub-micron patterns is important for ultrahigh-resolution QD-converted µ-LED displays, although this remains a challenge.

### µ-LED displays

Currently, many critical issues in µ-LED displays including the quantum-confined Stark effect, display resolution, pixel pitch, device EQE, colour conversion efficiency, and optical crosstalk have been relieved along with the development of nanofabrication capability and new structural design, such as super-inkjet printing, high-resolution lithography, advanced QD encapsulation, mould architecture design, and FRET nanostructure patterning. However, there are still some remaining obstacles for the high-throughput manufacturing of µ-LED displays.

Mass transfer is regarded as the greatest technical challenge in µ-LED display manufacturing. Damage should be avoided when the µ-LED is transferred from the mother wafer to the target substrate. However, rapid transfer can significantly increase the dead pixel number, resulting in a low manufacturing yield. The elastomer stamping approach can be applied to products of ~10 μm, with a high transfer yield of 99.99%;^61,62^ however, it takes a very long time to assemble a display (10 K µ-LED units per hour). Laser-assisted transfer can be applied to products of ~1 μm, with a high transfer speed (100 M µ-LED units per hour); however, the yield is only 90%^69^. Therefore, developing a stable and rapid mass transfer solution is the most pressing task to overcome the trade-off between production speed and pixel yield. For commercialisation of µ-LED displays, the target pixel yield is above 99.9999%. Thus, the dead pixel number can be controlled to less than 25 for a 4K display. However, it is still challenging to achieve such a high transfer yield. Another straightforward solution for pixel yield is transferring dual µ-LEDs into each pixel for illuminance backup^75^. However, the production cost of displays would be dramatically increased. Alternatively, individual pixel repair methods have been proposed to rescue dead pixels, such as laser welding, UV irradiation repair, selective rescue techniques, and redundant circuit design^70^. However, these approaches are also costly and not applicable for displays fabricated by monolithic integration.

Because the µ-LED display uses self-emission technology, a TFT matrix drive array is required to switch each pixel ON and OFF separately. The adoption of the TFT control matrix can significantly reduce the power consumption by ceasing unnecessary pixels and maintaining the operation of the display at low power. However, the small feature of µ-LEDs leads to the high spatial density of the TFT array and makes the circuit design more complicated. The bonding between the TFT matrix and µ-LED array is also problematic. Large-scale bonding with very small chip elements may cause short circuits due to the use of a large amount of metal in the solder paste.

Although obstacles exist, the current progress on µ-LED displays still provides an optimistic perspective for next-generation applications. According to UBI Research’s forecast, the market size of µ-LED displays will grow from around $100 million as of 2019 to over $6 billion by 2025, and the new emerging displays will widely be used in wearables, smartphones, TVs, and other applications^157^.

### Visible light communication using QD-converted µ-LED displays

Currently, the wireless network is in the era of a change driven by big data. Enormous growth in data-driven applications is triggering unprecedented demands on communication networks. However, the current wireless technology is primarily limited to the radio frequency (RF) band. Thus, the technology is no longer sufficient to support big data communications. Visible light communications (VLC) has become a promising technology to mitigate the RF spectrum crisis^158^. Different concepts for VLC are specified in standard IEEE 802.15.7. An interesting feature is the application of existing infrastructures such as LED displays to perform data transmission as transmitters. This approach supports a faster, safer, and more reliable wireless network for next-generation communications. As a result, VLC has been increasingly adapted for indoor optical wireless communication, underwater/seaside communication, in-vehicle data services, and the Internet of Things due to the outstanding advantages such as unregulated 260 THz modulation bandwidth in the wavelength range of 380–780 nm, high security and privacy by line-of-sight, lower cost and lower energy consumption^159^. Additionally, for the VLC using LED displays, due to multi-parallel transmission based on individual modulation of all pixels of the display, high data rates can be achieved. In VLC systems, conventional commercial LEDs with modulation bandwidths on the order of 10–20 MHz have been implemented for illumination, and the use of blue GaN µ-LEDs dramatically increases the bandwidth to several 100 MHz, with a data transfer rate of >1 Gbit/s. For example, Ferreira et al.^160^ demonstrated ultrahigh electrical-to-optical modulation bandwidths over 800 MHz with a blue GaN µ-LED. The transmission of data over free space at data rates of 1.7, 3.4, and 5 Gbit/s was demonstrated. Further, Islim et al. presented a data transmission rate of 11.95 Gbit/s with a GaN violet µ-LED by using a modulation scheme. The violet µ-LEDs were reported to have electrical-to-optical modulation bandwidths of 655 MHz^161^.

Recent demands of high-speed data transmission in VLC have motivated the adaptation of white lighting µ-LED arrays with pixel size approximately 100 µm, providing a peak data rate of >1 Gbit/s without complicated signal processing^160^. Thus, a white light source capable of wavelength multiplexing for VLC has become a vital issue, and enormous efforts have been devoted to increasing the modulation bandwidth by using phosphor-converted µ-LEDs. The white light produced by a blue GaN µ-LED and a yellow fluorescent polymer can be used to demonstrate a 1.68-Gb/s data transmission^162^. A commercial red RC-LED and custom green and blue µ-LEDs were used to achieve the aggregate highest data transmission of 11.28 Gb/s in white VLC light^162^. Recently, the generated white light from QDs on a blue GaN µ-LED has been explored for VLCs. As one example, the initial VLC was demonstrated with perovskite QD-converting µ-LEDs, and the results were ~160 MHz bandwidth at an emission wavelength of 445 nm and data rates of 300 Mbit/s^163^, which shows their great potential in the applications of high-speed and wide-bandwidth VLCs. Finally, we can expect to have cross-industry cooperation for the system integration of QD-converting µ-LED displays and VLCs to create a new commercial services value chain and a new wave of industry.

### Ultrahigh-definition displays and virtual reality/augmented reality displays

**Fig. 13:**
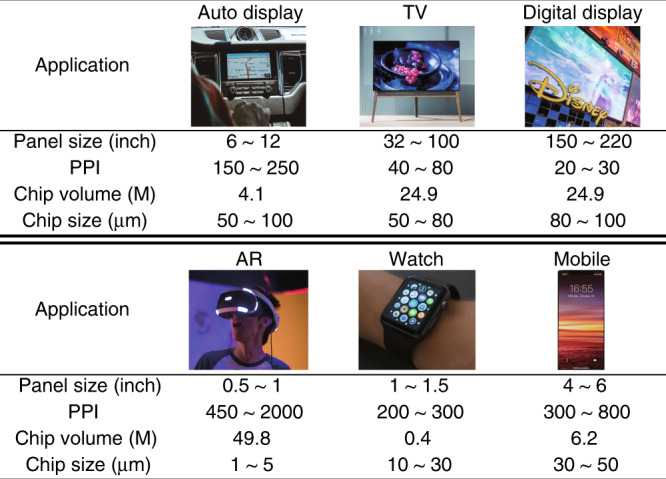
Requirements for µ-LEDs in typical applications. Reproduced from ref. ^35^ with permission from MDPI

µ-LEDs are suitable for advanced display applications such as smartphones, wearable watches, micro-projectors, AR/VR, automotive heads-up displays, and ultra HD (>4K resolution) televisions due to their advantages such as better colour accuracy, higher colour saturation, high contrast ratio, high peak brightness, low power consumption, and longer lifespan. Figure 13 shows the requirements for µ-LEDs in advanced display applications^35^.

In particular, QD-based µ-LED displays make a strong appeal to 8K ultra HD displays and AR/VR displays; 8K displays have a very high resolution of 7680 × 4320 and employ the Rec. 2020 system as a colour gamut standard. The most important feature of the Rec. 2020 standard is 99.9% reproducibility of colours in nature, and it leads to a drastically wider colour gamut than that of the HDTV standard (Rec. 709). Ultra HD displays require LEDs with better chromaticity in order to achieve a wider colour gamut as well as high efficiency and a long lifetime.

Moreover, displays for VR/AR applications require a light weight, small form factor, high pixel density, wide field-of-view (FoV), and high refresh rate to satisfy the human visual system. Generally, the FoV for each human eye is ~160° (horizontal) × 150° (vertical)^164^. Covering the full FoV with normal visual acuity (i.e., 60 pixels per degree) requires 9600 × 9600 pixels per eye, which is close to the resolution of ultra HD displays, and in 2018, Google demonstrated the highest resolution VR/AR OLED display, with 4800 × 3840 pixels per eye and an FoV of 120° × 96°^165^. In addition, avoiding screen flicker and reducing motion-to-photon latency requires the use of high refresh rates (>500 Hz) in displays, though it is quite impractical in current technologies (at present, 60–120 Hz). The high refresh rates and high resolution require a very fast pixel clock. For example, for the 9600 × 9000-pixel theoretical display with a 120-Hz refresh rate, the pixel clock is 93 GHz, corresponding to 10 ps. Of course, these conditions should be lowered to be realistic. Such a high resolution requires the pixel size to shrink down to the scale of a few µm, which results in a major challenge to have enough brightness on each colour because a single pixel has to be sub-divided into red, green and blue. The low optical efficiency of current AR/VR imaging optics cannot meet the bar for the minimum peak brightness. Specifically, for waveguide AR display, the overall efficiency can be <20%^166^. In such a case, the current OLEDs with a maximum luminance over 5000 nits (demonstrated by two research labs) cannot achieve an ambient contrast ratio of 5:1 (the contrast threshold of a recognisable image) in normal ambient lighting conditions^31,167^. Thus, the consensus is that QDs can provide the enough brightness under these conditions due to their high absorption cross-section and near-unity PLQY. µ-LEDs provide the device platform owing to their high efficiency, simple optics, and small form factor. Therefore, QD-based µ-LEDs (i.e., QD-converted or QD-EL µ-LEDs) could be the only viable technology for VR/AR displays.

Finally, µ-LEDs with flexible substrates represent a vital application, attracting enormous attention in the fields of displays, wearable electronics, lighting and biomedicine, and light guide plates because of their high efficiency, small form factor, and low power consumption. In particular, flexible and wearable µ-LED displays have thin, lightweight, and nonbreakable features, thus enabling the application of displays on curvilinear surfaces. Impressive colour purity and high brightness with low power consumption, high-resolution QD array patterning and ultrathin/ultrasmall form factors make QD-based µ-LEDs promising for flexible/wearable electronics.

## Conclusions

Colloidal QDs featuring narrow bandwidth emission and high PLQY provide promising advantages for their utilisation in display applications to create a wide colour gamut. The improved photostability of these QDs opens an opportunity for their utilisation in displays using optical excitation. Thus, a combination of rapid progress in device fabrication and performance of solid-state µ-LEDs and superior optical properties of QDs along with innovative fine-controlled QD synthesis and the development of favourable QD film deposition techniques can yield the most promising technology for next-generation displays. Of course, a fundamental understanding of the effects of the QD characteristics on their µ-LED display performance is necessary to reach this goal. Research into the stability and protection methods of QD films on µ-LEDs is still required to meet the strict stability requirements of commercial displays. Enormous efforts have been made in terms of the conversion efficiency and stability of QDs, especially considering their comparatively brief history in display applications. However, substantial further research into their encapsulation and large-scale patterning is imperative to enable commercial products. Nevertheless, the current progress on both µ-LEDs and QDs offers an optimistic perspective for potential implementation in next-generation displays.
